# Angiotensin II type 1a receptor loss ameliorates chronic tubulointerstitial damage after renal ischemia reperfusion

**DOI:** 10.1038/s41598-020-80209-0

**Published:** 2021-01-13

**Authors:** Yoko Fujita, Daisuke Ichikawa, Takeshi Sugaya, Keiichi Ohata, Jun Tanabe, Kazuho Inoue, Seiko Hoshino, Tatsuru Togo, Minoru Watanabe, Kenjiro Kimura, Yugo Shibagaki, Atsuko Kamijo-Ikemori

**Affiliations:** 1 Division of Nephrology and Hypertension, Department of Internal Medicine, St. Marianna University School of Medicine, 2-16-1 Sugao, Miyamae-Ku, Kawasaki, Kanagawa 216-8511 Japan; 2 Department of Anatomy, St. Marianna University School of Medicine, Kanagawa, Japan; 3 Institute for Animal Experimentation, St. Marianna University Graduate School of Medicine, Kanagawa, Japan; 4 JCHO Tokyo Takanawa Hospital, Tokyo, Japan

**Keywords:** Nephrology, Pathogenesis

## Abstract

We investigate whether suppressing the activation of the angiotensin II type 1a receptor (AT1a) can ameliorate severe chronic tubulointerstitial damage (TID) after renal ischemia reperfusion (IR) using AT1a knockout homozygous (AT1a^−/−^) male mice. To induce severe chronic TID after renal IR, unilateral renal ischemia was performed via clamping of the right renal pedicle in both AT1a^−/−^ and wild-type (AT1a^+/+^) mice for 45 min. While marked renal atrophy and severe TID at 70 days postischemia was induced in the AT1a^+/+^ mice, such a development was not provoked in the AT1a^−/−^ mice. Although the AT1a^+/+^ mice were administered hydralazine to maintain the same systolic blood pressure (SBP) levels as the AT1a^−/−^ mice with lower SBP levels, hydralazine did not reproduce the renoprotective effects observed in the AT1a^−/−^ mice. Acute tubular injury at 3 days postischemia was similar between the AT1a^−/−^ mice and the AT1a^+/+^ mice. From our investigations using IR kidneys at 3, 14, and 28 days postischemia, the multiple molecular mechanisms may be related to prevention of severe chronic TID postischemia in the AT1a^−/−^ mice. In conclusion, inactivation of the AT1 receptor may be useful in preventing the transition of acute kidney injury to chronic kidney disease.

## Introduction

The number of patients with chronic kidney disease (CKD) is increasing due to the aging populations of developed countries^1^. The progression of CKD leads not only to end stage renal disease (ESRD), but also to the onset of critical comorbidities, including cardiovascular diseases^2,3^. Useful treatments for the prevention of the progression of CKD are currently limited. The establishment of therapeutic strategies against the progression of CKD are therefore urgently required^4^.

The severity of tubulointerstitial damage (TID) is more closely associated with the renal prognosis of CKD than glomerular change^5^. Therefore, the management of TID is considered to slow CKD progression. Recently, it has been discovered that CKD can be induced after acute kidney injury (AKI)^6^. Although AKI was previously thought to be transient and reversible, accumulating evidence suggests that the transition from AKI to CKD can easily arise in elderly patients with decreased renal functional reserves due to aging^7–9^. Thus, various efforts to find a treatment to prevent the progression of CKD after AKI are currently underway^10,11^.

While the angiotensin II receptor blocker (ARB) contributes to the onset of AKI^12,13^, its effects on the prevention of transition from AKI to CKD have also been recognized^14,15^. However, its usefulness against marked severe TID after AKI has not yet been thoroughly investigated. Because the renal ischemic reperfusion (IR) model is generally used to reflect the pathology of ischemic AKI and subsequent CKD observed in clinical practice^16–18^, we investigated whether suppressed activation of the angiotensin II type 1a (AT1a) receptor could ameliorate severe chronic TID after renal IR using AT1a knockout homozygous (AT1a^−/−^) male mice. Although rodents have two isoforms of AT1 receptors, AT1a and AT1b, their isoforms are at two different loci that are 94% identical at the amino acid level and are pharmacologically indistinguishable from each other^19^. The AT1a receptor is the major mouse AT1 isoform and is expressed in most tissues, including the kidneys. AT1a plays a major role in the renal actions of Ang II^20^ and is the murine homologue to the single human AT1 receptor^21^. Therefore, AT1a^−/−^ male mice were used in the present study.

Our present study showed that defects in AT1a were related to the prevention of remarkable renal atrophy, attenuation of both interstitial fibrosis and chronic proximal tubular loss, but not to attenuation of acute tubular injury in a renal IR model. These results indicated that inactivation of the Ang II type 1 receptors may be effective in preventing the transition of AKI to CKD.

## Results

### AT1a receptor loss prevented AKI to CKD transition at 70 days postischemia

In our preliminary study, we found marked renal atrophy and severe chronic TID, which is similar to the renal pathology represented in human ESRD, at 70 days postischemia in a renal unilateral IR model of wild-type (AT1a^+/+^) mice with renal ischemia induced by clamping of the right renal pedicle for 45 min. Therefore, we investigated whether suppressed activation of the AT1a receptor could ameliorate severe TID in AT1a^−/−^ mice compared to AT1a^+/+^ mice at 70 days postischemia in the renal unilateral IR model. Furthermore, because our previous studies showed that the levels of systolic blood pressure (SBP) in AT1a^−/−^ mice were significantly lower than those in AT1a^+/+^ mice^22,23^, administration of hydralazine from 7 days before the IR operation to 70 days postischemia (AT1a^+/+^ + Hyd) was conducted in the AT1a^+/+^ mice to examine the influence of lower SBP levels on chronic TID postischemia.

#### Time-related changes in systolic blood pressure

**Figure 1:**
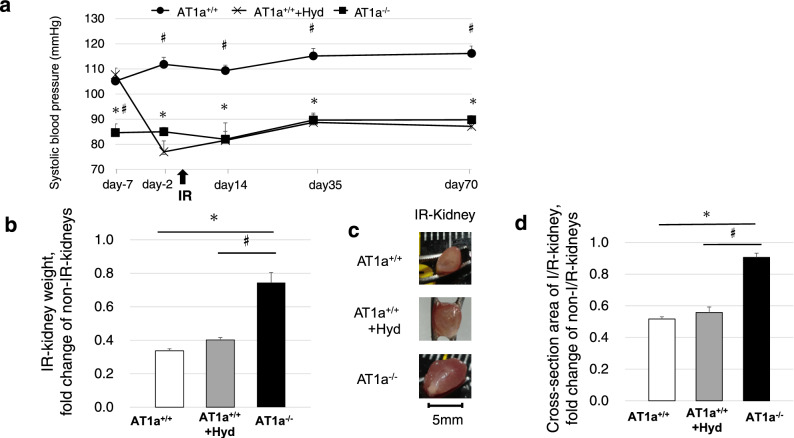
Effects of AT1a receptor loss on prevention of AKI to CKD transition at 70 days postischemia. The right kidneys received unilateral ischemic reperfusion (IR) and the left kidneys were contralateral (non-IR kidneys). (**a**) Time-related changes in systolic blood pressure from 7 days before IR surgery to 70 days postischemia. (**b**) IR kidney weight shown as the fold-increase or fold-decrease compared with the non-IR kidneys. (**c,d**) Cross-sectional area of the IR kidney cut in transverse plane at the hilum of the kidney (**c**), which was evaluated as the fold-increase or fold-decrease compared with the non-IR kidney (**d**). Values are means ± SE*.* *P < 0.05 vs. AT1a^+/+^ mice on the same day, ^#^*P* < 0.05 vs. the AT1a^+/+^  + Hyd mice on the same day.

SBP levels were significantly lower in the AT1a^−/−^ mice compared to those in the AT1a^+/+^ mice from 7 days before the IR operation until 70 days postischemia (*p* < 0.05, Fig. 1a). SBP levels in AT1a^+/+^ + Hyd mice were decreased to the same levels as the AT1a^−/−^ mice two days before the IR operation and these lower levels were maintained until 70 days postischemia (*p* < 0.05, Fig. 1a).

#### Weight and cross-section area of IR kidney

The weight (Fig. 1b) and the cross-sectional area of the IR kidney (Fig. 1c) of both AT1a^+/+^ and AT1a^+/+^ + Hyd mice were extremely low compared to the contralateral kidney (non-IR kidney), and these lows were significantly inhibited in the AT1a^−/−^ mice at 70 days postischemia (*p* < 0.05, Fig. 1b–d).

#### Renal immunohistological analysis of tubulointerstitial damage

**Figure 2:**
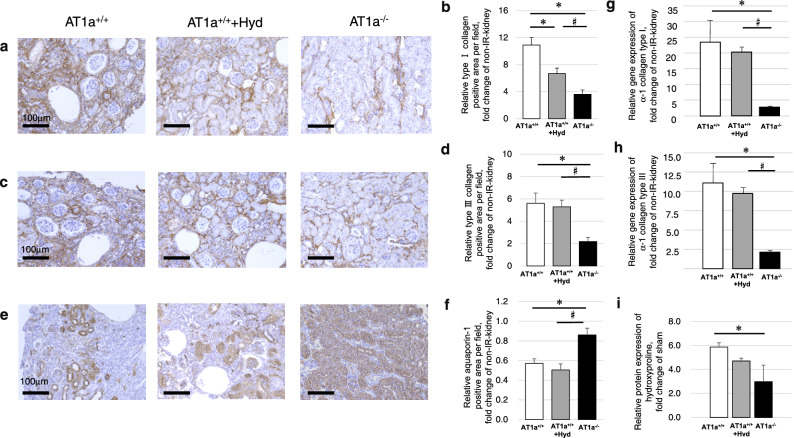
Chronic tubulointerstitial damage at 70 days postischemia. (**a,c,e**) Immunohistochemical staining of type I collagen (**a**), type III collagen (**c**), and aquaporin-1 as a proximal tubular marker (**e**) in the IR kidneys. (**b,d,f**) Quantification of each positively stained area of type I collagen (**b**), type III collagen (**d**), and aquaporin-1 (**f**). Original magnification, × 100. (**g,h**) Gene expressions of α-1 collagen type I (**g**) and α-1 collagen type III (**h**). (**i**) Renal levels of hydroxyproline. The graphs show the fold-increase or -decease in each positively stained area or each gene expression in the IR kidney compared with the contralateral kidney (non-IR kidney). Values are means ± SE*.* *P < 0.05 vs. AT1a^+/+^ mice on the same day, ^#^*P* < 0.05 vs. AT1a^+/+^  + Hyd mice on the same day.

In order to examine the degree of interstitial fibrosis, the expressions of type I and type III collagens were determined by immunohistochemical analysis. The depositions of type I collagen (Fig. 2a,b) and type III collagen (Fig. 2c,d) were increased in the interstitia of the IR kidneys compared to the non-IR kidneys in all mice and were significantly lower in the AT1a^−/−^ mice than in both the AT1a^+/+^ and AT1a^+/+^ + Hyd mice at 70 days postischemia (*p* < 0.05). Although the deposition levels of type I collagen in the AT1a^+/+^ + Hyd mice were significantly lower than in the AT1a^+/+^ mice (*p* < 0.05, Fig. 2b), the deposition levels of type III collagen were similar between the AT1a^+/+^ and AT1a^+/+^ + Hyd mice (Fig. 2d).

For evaluation of the remaining proximal tubules, aquaporin-1 was immunohistochemically detected and analyzed (Fig. 2e,f). Remarkable loss of aquaporin-1-positive areas in the IR kidneys were observed in both AT1a^+/+^ and AT1a^+/+^ + Hyd mice, and aquaporin-1-positive areas were significantly greater in the AT1a^−/−^ mice compared to the AT1a^+/+^ and AT1a^+/+^ + Hyd mice at 70 days postischemia (*p* < 0.05, Fig. 2f).

#### Gene expression analysis and hydroxyproline assay for renal fibrosis

The gene expressions of *α-1 collagen type I* (Fig. 2g) and *α-1 collagen type III* (Fig. 2h) were upregulated in the IR kidneys compared to non-IR kidneys in all mice, and their levels in the AT1a^−/−^ mice were significantly lower compared to the AT1a^+/+^ and AT1a^+/+^ + Hyd mice at 70 days postischemia (*p* < 0.05). As fibrillar collagens of all types contain hydroxyproline, renal hydroxyproline was evaluated for renal fibrosis (Fig. 2i). The degree of renal hydroxyproline increased in the IR kidneys compared to non-IR kidneys in the AT1a^+/+^, AT1a^+/+^ + Hyd, and AT1a^−/−^ mice (p < 0.05). The degree in the AT1a^−/−^ mice was significantly lower compared to the AT1a^+/+^ mice (*p* < 0.05) and tended to be lower than that in the AT1a^+/+^ + Hyd mice (p = 0.08). The degree was similar between the AT1a^+/+^ mice and the AT1a^+/+^ + Hyd mice.

### Evaluation of AKI at 3 days postischemia

We compared the degree of acute tubular injury postischemia between AT1a^−/−^ and AT1a^+/+^ mice because the initial seriousness of tubular injury induced by IR is strongly related to the degree of ensuing chronic TID^17^. Because there is a possibility that an IR kidney has an influence on the contralateral kidney^24^, sham operated mice were used as a control.

#### Weight and cross-section area of IR kidney

**Figure 3:**
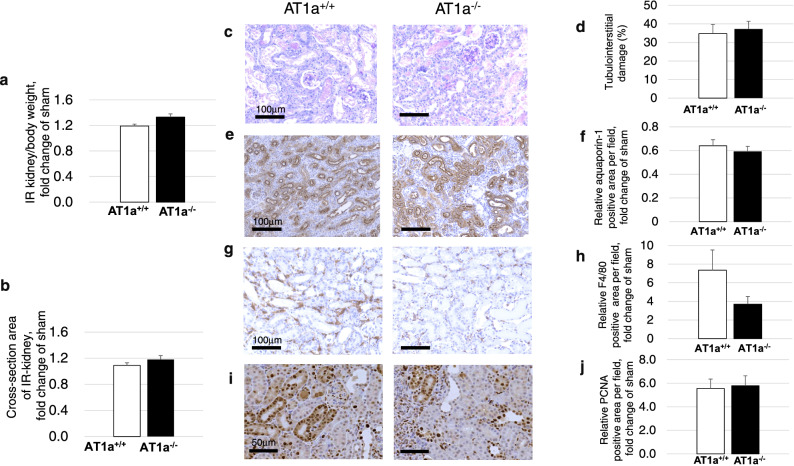
Effects of AT1a receptor loss on AKI severity at 3 days postischemia. The right kidneys received unilateral ischemic reperfusion (IR) and sham mice were treated in a similar manner without clamping of the renal pedicle. (**a**) IR kidney weight-to-body weight ratio shown as the fold-increase or fold-decrease compared with that of sham. (**b**) Cross-sectional area of the IR kidney cut in transverse plane at the hilum of the kidney, which was evaluated as the fold-increase or fold-decrease compared with that of the sham. (**c,d**) Histologic analyses for tubulointerstitial damage in tissues stained with periodic acid-Schiff (PAS) in the IR kidneys (**c**). Tubulointerstitial damage in the IR kidneys was assessed quantitatively in the PAS-stained sections (**d**). (**e,f**) Immunohistochemical staining (**e**) and quantification of the positively stained areas of aquapolin-1 in the IR kidneys (**f**). (**g,h**) Immunohistochemical staining (**f)** and quantification of the positively stained areas of F4/80 in the IR kidneys (**h**). (**i,j**) Immunohistochemical staining (**i**) and quantification of the positively stained areas of proliferating cell nuclear antigen (PCNA) in the IR kidneys (**j**). The graph shows the fold-increase or -decease in positively stained areas of aquapoline-1, F4/80, and PCNA in the IR kidney compared with that of the sham. Original magnification, × 100 for PAS, aquapoline-1 and F4/80, and × 200 for PCNA. Values are means ± SE.

Renal swelling was observed at 3 days postischemia in the IR kidneys of both AT1a^−/−^ and AT1a^+/+^ mice. Both the IR kidney weight to body weight ratio (Fig. 3a) and the cross-sectional area of the IR kidney (Fig. 3b) were similarly increased in both AT1a^−/−^ and AT1a^+/+^ mice at 3 days postischemia.

#### Renal histological analysis

Acute tubular injury was defined as tubular necrosis, brush-border loss, cast formation, tubular dilation, and tubular degeneration in periodic acid-Schiff (PAS)-stained sections (Fig. 3c) and was observed in the IR kidneys of both AT1a^−/−^ and AT1a^+/+^ mice. The degree of acute tubular changes in the IR kidneys was similar in both types of mice at 3 days postischemia (Fig. 3d).

#### Renal immunohistological analysis of tubulointerstitial damage and for evaluation of tubular cell proliferation

For evaluation of tubulointerstitial damage of the kidney, aquapolin-1-positive cells were immunohistochemically detected and analyzed (Fig. 3e,f). Aquaporin-1-positive areas in IR kidneys were similarly decreased in both AT1a^+/+^ and AT1a^−/−^ mice compared to the kidneys of the sham mice. For evaluation of macrophage infiltration of the kidney, F4/80-positive cells were immunohistochemically detected and analyzed (Fig. 3g,h). The F4/80-positive areas in the interstitia of the IR kidneys were similarly increased in both AT1a^+/+^ and AT1a^−/−^ mice compared to the kidneys of the sham mice (p = 0.13). For evaluation of tubular cell proliferation, proliferating cell nuclear antigen (PCNA) was immunohistochemically detected and analyzed (Fig. 3i,j). PCNA-positive areas were observed in the nuclei of tubular cells. The expression levels of PCNA in the IR kidneys were similarly increased in both AT1a^−/−^ and AT1a^+/+^ mice (Fig. 3j).

#### Gene expression analysis for renal inflammatory and profibrotic cytokines

**Figure 4:**
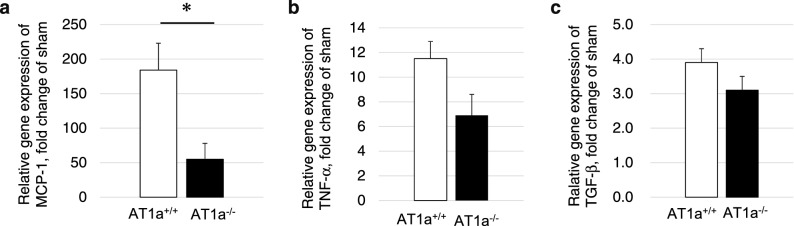
Gene expression analysis for renal inflammatory and pro-fibrotic cytokines at 3 days postischemia. (**a–c**) Gene expressions of *MCP-1* (**a**), *TNF-*α (**b**), and *TGF-*β (**c**) in the IR kidneys. The graphs show the fold-increase or -decease in each gene expressions in the IR kidney compared with that of the sham. Values are means ± SE*.* *P < 0.05 vs. AT1a^+/+^ mice on the same day.

To evaluate inflammatory and profibrotic responses, the renal gene expressions of *MCP-1* (Fig. 4a)*, tumor necrosis factor-α (TNF-α)* (Fig. 4b), and transforming growth factor-β (*TGF-*β (Fig. 4c) were measured. The gene expression levels of *MCP-1, TNF-α, and TGF-*β in the IR kidneys at 3 days postischemia were upregulated in both mutant and wild-type mice compared to the kidneys of sham mice. In the IR kidneys at 3 days postischemia, the gene expression levels of MCP-1 were significantly lower (*p* < 0.05, Fig. 4a) and the gene expression levels of TNF-a tended to be lower (p = 0.07, Fig. 4b) in the AT1a^−/−^ mice than in the AT1a^+/+^ mice. Regarding TGF-b, the gene expression levels were similar in both types of mice (Fig. 4c).

### Mechanism for renoprotection of AT1a receptor loss against AKI to CKD transition

Because the observed chronic renal morphology with marked atrophy and severe TID at 70 days postischemia in AT1a^+/+^ mice was close to the final pathology of CKD, we evaluated the TID at 14 and 28 days postischemia in order to clarify the role of AT1a receptor loss on AKI-to-CKD transition. Sham mice were used as a control.

#### Weight and cross-section area of IR kidney

**Figure 5:**
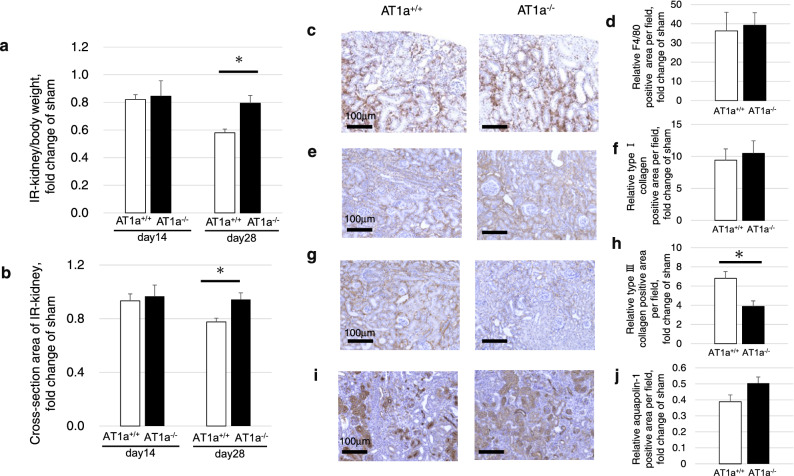
Effects of AT1a receptor loss on prevention of AKI to CKD transition at 14 and 28 days postischemia. (**a**) IR kidney weight-to-body weight ratio shown as the fold-increase or fold-decrease compared with that of sham at 14 and 28 days postischemia. (**b**) Cross-sectional area of the IR kidney cut in transverse plane at the hilum of the kidney at 14 and 28 days postischemia, which was evaluated as the fold-increase or fold-decrease compared with that of the sham. (**c,e,g,i**) Immunohistochemical staining of F4/80 (**c**), type I collagen (**e**), type III collagen (**g**), and aquaporin-1 (**i**) in the IR kidneys at 14 days postischemia. (**d,f,h,j**) Quantification of the positively stained areas of F4/80 (**d**), type I collagen (**f**), type III collagen (**h**), and aquaporin-1 (**j**) in the IR kidneys at 14 days postischemia. The graphs show the fold-increase or -decease in each positively stained area in the IR kidney compared with that of the sham. Original magnification, × 100. Values are means ± SE*.* *P < 0.05 vs. AT1a^+/+^ mice on the same day.

While both the IR kidney weight to body weight ratio (Fig. 5a) and the cross-sectional area of the IR kidney (Fig. 5b) in the AT1a^−/−^ mice were similar to those in AT1a^+/+^ mice at 14 days postischemia, these levels in the AT1a^+/+^ mice decreased and were significantly lower than in the AT1a^−/−^ mice (*p* < 0.05) at 28 days postischemia.

#### Renal immunohistological analysis of tubulointerstitial damage

For evaluation of macrophage infiltration of the kidney, F4/80-positive cells were immunohistochemically detected and analyzed (Fig. 5c,d). The F4/80-positive areas in the interstitia of the IR kidneys similarly increased compared to the kidneys of sham mice in both mutant and wild-type mice at 14 days postischemia. The deposition levels of type I and type III collagen on the interstitia of the IR kidneys increased in both mutant and wild-type mice compared to sham mice. Although the deposition levels of type I collagen in the IR kidneys were similar between AT1a^+/+^ and AT1a^−/−^ mice at 14 days postischemia (Fig. 5e,f), the deposition levels of type III collagen were significantly lower in the AT1a^−/−^ mice compared to the AT1a^+/+^ mice at 14 days postischemia (Fig. 5g,h). Regarding the expression of aquaporin-1 in IR kidneys, decreases in aquaporin-1-positive areas in IR kidneys were similarly observed in both mutant and wild-type mice compared to the kidneys of the sham mice at 14 days postischemia (Fig. 5i,j).

At 28 days postischemia, although the F4/80-positive areas (Supplementary Fig. S1a,b), the deposition levels of type I (Supplementary Fig. S1c,d) and type III (Supplementary Fig. S1e,f) collagens in the IR kidneys increased in both mutant and wild-type mice compared to the kidneys of the sham mice, their levels in the AT1a^−/−^ mice were significantly lower than in the AT1a^+/+^ mice (*p* < 0.05). Similarly, the decreases in aquaporin-1-positive areas in IR kidneys in both mutant and wild-type mice compared to the kidneys of the sham mice were significantly lower in the AT1a^−/−^ mice than in the AT1a^+/+^ mice (*p* < 0.05, Supplementary Fig. S1g,h).

#### Gene expression analysis for renal fibrosis

**Figure 6:**
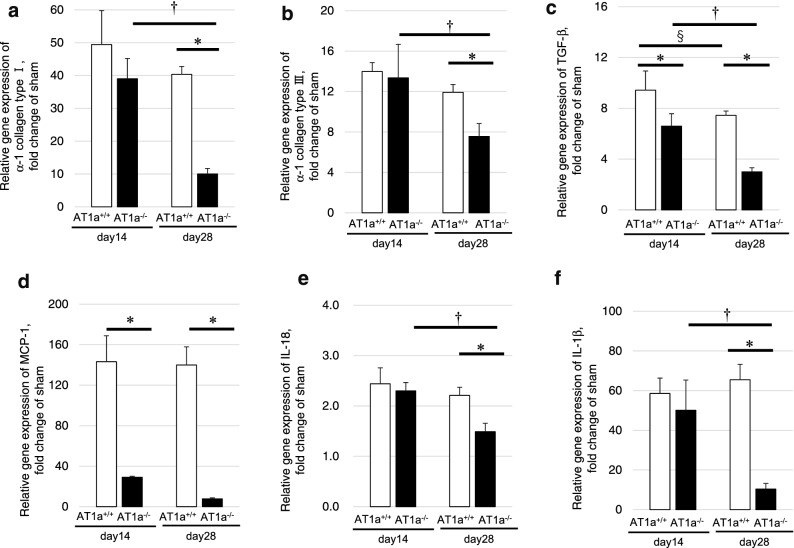
Gene expression analysis for renal fibrosis and inflammatory cytokines at 14 and 28 days postischemia. (**a–f**) Gene expressions of α*-1 collagen type I* (**a**), α*-1 collagen type III* (**b**), *TGF-*β (**c**), *MCP-1* (**d**), *IL-18* (**e**), and *IL-1*β (**f**) in the IR kidneys. The graphs show the fold-increase or -decease in each gene expression in the IR kidney compared with that of the sham. Values are means ± SE*.* *P < 0.05 vs. AT1a^+/+^ mice on the same day, ^§^*P* < 0.05 vs. AT1a^+/+^ mice at 14 days postischemia, ^†^*P* < 0.05 vs. AT1a^−/−^ mice at 14 days postischemia.

The gene expression levels of *α-1 collagen type I* (Fig. 6a) and *α-1 collagen type III* (Fig. 6b) were upregulated in the IR kidneys compared to the kidneys of the sham mice at 14 days and 28 days postischemia in both mutant and wild-type mice. While the gene expression levels of *α-1 collagen type I* (Fig. 6a) and *α-1 collagen type III* (Fig. 6b) in the IR kidneys at 14 days postischemia were similar between the AT1a^+/+^ and the AT1a^−/−^ mice, the gene expression levels in the AT1a^−/−^ mice significantly decreased at 28 days postischemia, and were significantly lower than in the AT1a^+/+^ mice (*p* < 0.05).

The gene expression levels of transforming growth factor-β (*TGF-*β), a profibrotic marker, in the IR kidneys at 14 and 28 days postischemia were upregulated in both mutant and wild-type mice compared to in the kidneys of sham mice, but were significantly lower in the AT1a^−/−^ mice compared to the AT1a^+/+^ mice (*p* < 0.05, Fig. 6c). The expression level of *TGF-*β in the IR kidneys of both the AT1a^+/+^ and the AT1a^−/−^ mice was significantly decreased at 28 days postischemia compared to 14 days postischemia (*p* < 0.05, Fig. 6c).

#### Gene expression analysis for renal inflammatory cytokines

To evaluate inflammatory responses, the renal gene expressions of monocyte chemoattractant protein-1 *(MCP-1)* (Fig. 6d), interleukin-18 *(IL-18)* (Fig. 6e), and *IL-1*β (Fig. 6f) were measured. The gene expression levels of *MCP-1, IL-18, and IL-1*β in the IR kidneys at 14 and 28 days postischemia were upregulated in both mutant and wild-type mice compared to in the kidneys of sham mice. The gene expression levels of *MCP-1* in the IR kidneys at 14 and 28 days postischemia were significantly lower in the AT1a^−/−^ mice than in the AT1a^+/+^ mice (*p* < 0.05, Fig. 6d). Although the gene expression levels of *IL-18* (Fig. 6e) and *IL-1*β (Fig. 6f) in the IR kidneys at 14 days postischemia were similar between AT1a^+/+^ and AT1a^−/−^ mice, their levels at 28 days postischemia were significantly lower in the AT1a^−/−^ mice than in the AT1a^+/+^ mice (*p* < 0.05, Fig. 6e,f). In the AT1a^−/−^ mice, their levels were significantly decreased at 28 days postischemia compared to 14 days postischemia (*p* < 0.05, Fig. 6e,f).

#### Evaluation of renal oxidative stress, hypoxia, and CD34 protein expression

**Figure 7:**
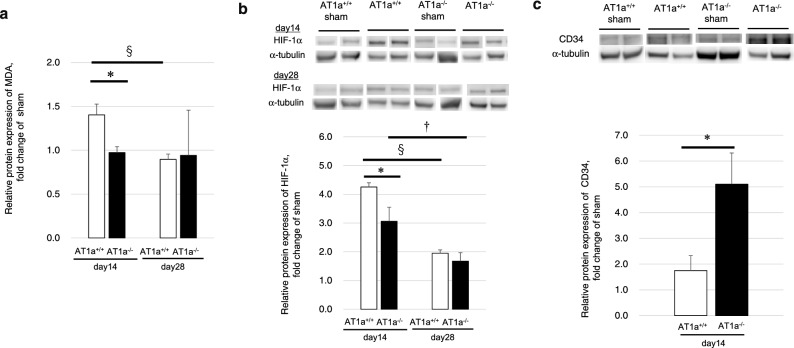
Evaluation of renal oxidative stress and hypoxia at 14 and 28 days postischemia and CD34 protein expression at 14 days postischemia. (**a**) Protein expression of malondialdehyde (MDA) at 14 and 28 days postischemia in the IR kidneys. (**b**) Western blot analysis of renal HIF-1α and α-tubulin in the IR kidneys at 14 and 28 days postischemia. (**c**) Western blot analysis of CD34 and α-tubulin in the IR kidneys at 14 days postischemia. The representative blots were shown by cropping from different parts of the same bolt for HIF-1α and CD 34. The full-length blots are presented in Supplementary Fig. S5. The graphs show the fold-increase or -decease in each protein expression in the IR kidneys compared with that of the sham. Values are means ± SE*.* *P < 0.05 vs. AT1a^+/+^ mice on the same day, ^§^*P* < 0.05 vs. AT1a^+/+^ mice at 14 days postischemia, ^†^*P* < 0.05 vs. AT1a^−/−^ mice at 14 days postischemia.

To evaluate the degree of renal oxidative stress, renal malondialdehyde (MDA) levels were measured. Renal protein levels of MDA in the IR kidneys at 14 days postischemia in the AT1a^+/+^ mice increased and were significantly higher than in the AT1a^−/−^ mice (*p* < 0.05, Fig. 7a). At 28 days postischemia, the mean values of renal MDA in the IR kidneys of the AT1a^+/+^ mice were significantly decreased compared to14 days postischemia and were similar to the AT1a^−/−^ mice (Fig. 7a).

Hypoxia inducible factor-1α (HIF-1α) is upregulated under renal hypoxia. The protein expression levels of HIF-1α in the IR kidneys at 14 days postischemia were increased in both mutant and wild-type mice compared to those of sham mice. While the HIF-1α expression levels in the IR kidneys at 14 days postischemia were significantly higher in the AT1a^+/+^ mice than in the AT1a^−/−^ mice (*p* < 0.05, Fig. 7b), the renal HIF-1α expression in the IR kidneys at 28 days postischemia were significantly decreased compared to 14 days postischemia and were similar between AT1a^+/+^ and AT1a^−/−^ mice (*p* < 0.05, Fig. 7b).

Because renal hypoxia, evaluated by renal protein expression of HIF-1α, was strongly induced in the IR kidneys at 14 days postischemia in the AT1a^+/+^ mice compared to the AT1a^−/−^ mice, the renal protein expression of CD34, a marker of endothelial cells and endothelial progenitor cells, was examined in the IR kidneys at 14 days postischemia. The CD34 expression levels were increased in the IR kidneys of the AT1a^−/−^ mice compared to the kidneys of sham mice and the levels were significantly higher than in the AT1a^+/+^ mice (*p* < 0.05, Fig. 7c).

#### Expression analyses of Wnt/β-catenin

**Figure 8:**
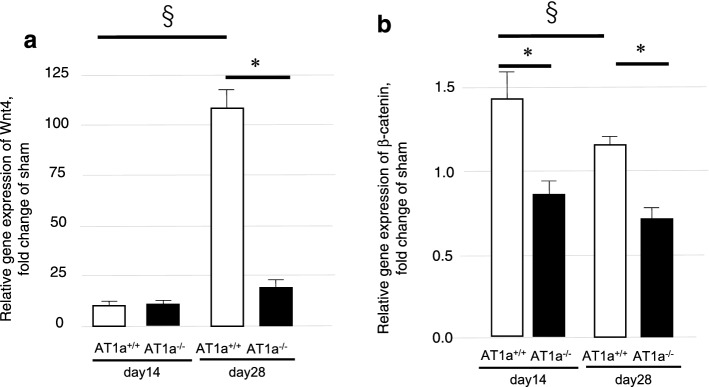
Expression analyses of Wnt/β-catenin at 14 and 28 days postischemia. (**a,b**) Gene expressions of *Wnt4* (**a**) and β*-catenin* (**b**) in the IR kidneys at 14 and 28 days postischemia. The graphs show the fold-increase or -decease in each gene and protein expression in the IR kidneys compared with that of the sham. Values are means ± SE. *P < 0.05 vs. AT1a^+/+^ mice on the same day, ^§^*P* < 0.05 vs. AT1a^+/+^ mice at 14 days postischemia.

Because sustained activation of Wnt/β-catenin signaling has been reported to be a driving force for AKI-to-CKD transition^25^, the expressions of *Wnt 4* (Fig. 8a) and β*-catenine* (Fig. 8b) were evaluated in the IR kidneys. While the Wnt4 expression levels in the IR kidneys at 14 days postischemia were similar between mutant and wild-type mice, the levels at 28 days postischemia were significantly increased in the AT1a^+/+^ mice compared to 14 days postischemia and were significantly higher in the AT1a^+/+^ mice than in the AT1a^−/−^ mice at 28 days postischemia (*p* < 0.05, Fig. 8a).

The gene expression meant that levels of β*-catenin* in the IR kidneys of the AT1a^+/+^ mice at 14 and 28 days postischemia were significantly higher than in the AT1a^−/−^ mice (*p* < 0.05, Fig. 8b). The β*-catenin* gene expression levels in the IR kidneys were significantly decreased at 28 days postischemia compared to at 14 days postischemia in the AT1a^+/+^ mice (*p* < 0.05, Fig. 8b).

## Discussion

The present study demonstrates that marked renal atrophy and notable chronic TID with higher expressions of both type I and type III collagens at 70 days postischemia induced in AT1a ^+/+^ mice were significantly ameliorated in AT1a^−/−^ mice. Although the AT1a^+/+^ mice were administered hydralazine to maintain the same systolic blood pressure (SBP) levels as the AT1a^−/−^ mice with lower SBP levels, hydralazine did not reproduce the renoprotective effects observed in the AT1a^−/−^ mice. Acute tubular injury, the loss of proximal tubules, macrophage infiltration and the degree of tubular cell proliferation at 3 days postischemia were similar between the AT1a^−/−^ mice and the AT1a^+/+^ mice. From our investigations using IR kidneys, the underlying molecular mechanism for the prevention of severe chronic postischemic TID in the AT1a^−/−^ mice was considered to be due to anti-inflammation, anti-oxidation, anti-hypoxia, inactivation of Wnt/β-catenin signaling, and retention of AT2 receptor expression. These results indicate that inactivation of the Ang II type 1 receptor might be useful for the prevention of the transition of AKI to CKD.

Activation of the AT1a receptor plays a central role in the progression of CKD through the acceleration of oxidative stress via activation of NAD(P)H oxidase, which causes renal inflammation and fibrosis^28^. This study demonstrates that a decrease in renal MDA levels produced by oxidative stress at 14 days postischemia via the downregulation of *p47-phox(NCF1)*, a subunit of NAD(P)H oxidase (Supplementary Fig. S2), and the prevention of interstitial inflammation via the downregulation of inflammatory cytokines, *MCP-1* (14 and 28 days postischemia) and *IL-18* and *IL-1*β (28 days postischemia) lead to the prevention of interstitial fibrosis in mice that lack the AT1a receptor. However, the loss of AT1a receptor expressed on macrophages and T lymphocytes infiltrated the renal interstitium has been reported to be associated with acute toxic renal injury and progression of renal fibrosis via generation of inflammatory cytokines^29,30^. Mice lacking the systemic AT1a receptor were used in our study, and the effect of AT1a receptor loss on the tubular epithelium, a target of IR injury, may be related to a reduction in oxidative stress and generation of inflammatory cytokines after IR, thereby preventing AKI-to-CKD transition following lessened infiltration of inflammatory cells and interstitial fibrosis.

Renal IR injury induces rarefaction of peritubular capillaries following tubular injury and provokes tubular hypoxia, resulting in AKI-to-CKD transition^31^. Because the expression of renal HIF-1α increased along with the severity of renal hypoxia^17^, the degree of chronic tubular hypoxia after IR was evaluated via the renal expressions of HIF-1α. As a result, the mice without the AT1a receptor showed suppression of upregulated renal HIF-1α expression at 14 days postischemia and upregulation of CD34 expression. These results indicate that the AT1a receptor loss mitigated the induction of tubular hypoxia via retention of peritubular capillaries and recovery of damaged peritubular capillaries by attenuation of chronic tubular injury after IR.

Contrary to our results, the upregulated expression of renal HIF-1α by AT1 receptor blocker accompanied by attenuation of CKD after IR was previously reported in rats^14^. These conflicting results can be attributed to the several differences between the studies, including species, initial severity of IR injury, and IR model (bilateral or unilateral IR). In our further analysis using mice with AT1a receptors, renal HIF-1α expression increased at 14 days postischemia but thereafter decreased. At 28 days postischemia, renal HIF-1α expression decreased to the same level as the mice without AT1a receptor, suggesting that upregulation of renal HIF-1α expression was transient. Although there is a possibility that CKD after IR was too severe to maintain the upregulation of renal HIF-1α expression in the mice with AT1a receptors, further study is required to clarify how transient changes in renal HIF-1α expression after IR has an influence on the pathophysiology of AKI-to-CKD transition.

Upregulation of renal *Wnt4* and β*-catenin* expressions at 28 days postischemia was observed in the mice with AT1 receptors. Renal *Wnt4* expression has been reported to increase after postischemia and sustained activation of Wnt/β-catenin signaling was reported to become the driving force for AKI-to-CKD transition^25^. Our results were consistent with the previous study. Furthermore, AT1 receptor-dependent activation of β-catenin signaling has been reported to cause renal fibrosis^32,33^, and such a mechanism in the mice with AT1a receptors may be related to the upregulation of β*-catenin* gene expression without an increase in *Wnt4* gene expression compared to the mice without AT1a receptors at 14 days postischemia. Inactivation of Wnt/β-catenin signaling via AT1a receptor loss may contribute to the prevention of severe CKD after IR.

The mice with AT1a receptor loss used in our study retained the expression of the Ang II type 2 (AT2) receptor and higher gene expression levels of the *AT2 receptor* were observed in the IR kidneys of the mice without AT1a receptors compared to mice with AT1a receptor at 28 days postischemia (Supplementary Fig. S3). Although the severity of chronic TID may affect the different expression levels of the renal *AT2 receptor*, the anti-fibrotic function of the AT2 receptor via disruption of the TGF-β signaling pathway has been reported in a progressive renal fibrosis model and a unilateral ureteral obstruction model, using AT2 receptor deficient mice^26,27^. The greater *AT2 receptor* expression in the mice without AT1a receptors may be partly related to the downregulation of *TGF-*β gene expression at 28 days postischemia. There is a possibility that maintenance of AT2 receptor expression in the mutant mice contributed to the amelioration of severe CKD after IR. Further study is required to clarify the role of the AT2 receptor in AKI-to-CKD transition in mice without AT1a receptors.

Previous studies have reported the protective effects of the AT1 receptor blocker against acute tubular injury due to IR^34,35^. Although the present study showed inhibited renal gene expression of inflammatory cytokine due to AT1a receptor loss, acute renal structural changes, including tubular necrosis at 3 days postischemia, were strongly provoked in our model compared to the previous report^35^. Amelioration of acute tubular damage, inhibition of proximal tubular loss and macrophage infiltration, and acceleration of tubular cell proliferation was not observed in mice without AT1a receptors compared to mice with AT1a receptor. Because the deficiency of AT1a receptor signaling on T lymphocytes has been reported to be related to aggravation of acute tubular injury, as described above^30^, inactivation of the AT1a receptor in T lymphocytes may contribute to severe acute tubular injury after IR in mice without AT1a receptors. Furthermore, in clinical practice, perioperative hypotension has been reported to be associated with the onset of postoperative AKI^36,37^, indicating that hypotension impairs renal perfusion, leading to the development of AKI^38^. We also found that lower SBP with hydralazine, commonly used as a vasodilator, led to acceleration of proximal tubular loss and up-regulation of pro-fibrotic cytokine gene expression (Supplementary Fig. S4). The significantly lower SBP levels noted in mice without AT1a receptors may sustain renal hypoperfusion and subsequently provoke similar degree of acute tubular damage after IR to the mice with AT1a receptor in spite of down-regulation of inflammatory cytokine due to AT1a receptor loss.

While hypotension in the acute phase after IR may aggravate acute renal injury, hypertension is a risk factor for the progression of CKD^39,40^. Because mice without AT1a receptors have sustained lower SBP, it was considered that lower SBP may prevent severe chronic renal injury after IR. However, the renoprotective effect of AT1a receptor loss was not completely reproduced by lower SBP with hydralazine. On the other hand, we did observe a decrease in renal deposition of type I collagen by hydralazine. Treatment with hydralazine has been reported to prevent renal fibrogenesis after IR via the demethylation of the methylated promotor region of *RASAL1*, which encodes RAS-Gap-like protein 1 and is related to the inactivation of fibroblasts, independent of its blood pressure-lowing effects^41^. In our model, the demethylating activity of hydralazine may have partially contributed to the prevention of renal fibrosis in the wild-type mice that were injected with hydralazine.

The results of this study may have been limited by some factors. First, the favorable effect of AT1a receptor loss for chronic renal dysfunction after AKI was not evaluated in order to use the unilateral IR model with a longer ischemic time for evaluating the effect of the AT1a receptor loss against accelerated AKI-to-CKD transition. Second, because of inhibited expression of inflammatory cytokine due to AT1a receptor loss in the acute phase after IR, the preoperative use of RAS inhibitors might be important for the prevention of severe AKI to CKD transition. However, there is concern that the preoperative usage of RAS inhibitors increases the risk of AKI onset due to decreased glomerular filtration especially in elderly patients with lower renal functional reserve. Further clinical analysis is needed to determine which generation can benefit from the effects of RAS inhibitors for prevention of severe AKI-to-CKD transition through their preoperative use.

In conclusion, the findings of this study reveal that AT1a receptor loss prevented AKI-to-CKD progression via various pathways. Recent clinical studies have shown that RAS inhibitors, started during the recovery phase after AKI, reduce the incidence of CKD^42^. The use of RAS inhibitors in patients who experience AKI does not increase the risk of recurrent AKI^43^ and decreases the one-year mortality^44^. Therefore, the use of RAS inhibitors may contribute to a decrease in the occurrence of CKD after AKI and improve the prognosis of patients with AKI. Our preclinical results highlight the usefulness of the inactivation of the Ang II type 1 receptor for the prevention of AKI-to-CKD transition.

## Method

### Animals

This study was conducted according to St. Marianna University School of Medicine Institutional Guide for Animal Experiments and the Guide for the Care and Use of Laboratory Animals (National Institutes of Health, Bethesda, MD, USA). All the experimental protocols in this study were approved by the Ethical Committee on Animal Experiments of St. Marianna University School of Medicine (TG200325-4C). Male C57/BL6 wild-type (AT1a^+/+^) mice were purchased from Japan SLC (Shizuoka, Japan). AT1a receptor knockdown homozygous (AT1a^−/−^) mice from a C57/BL6 background were generated as described previously^45^. Eight- to ten-week-old male mice (weight, 22–27 g) were used for the experiments. All mice had free access to water and were fed regularly.

### Experimental design

Renal ischemia was induced by clamping of the right renal pedicle with a cerebral aneurysm clip for 45 min after right dorsal laparotomy under inhalation anesthesia with 2% isoflurane, followed by reperfusion. In a preliminary study to establish a model with both severe TID and renal atrophy after IR, we found that approximately 90% of male mice that were clamped for either 25 or 30 min with unilateral nephrectomy died within 7 days. Furthermore, clamping for 20 min induced interstitial fibrosis, but not renal atrophy in the chronic phase. Therefore, the unilateral long-term (45 min) IR model without unilateral nephrectomy was used in the present study. IR mice were sacrificed immediately after euthanasia by intraperitoneal anesthesia with 400 mg/kg pentobarbital at 3 days (AT1a^+/+^, n = 9; AT1a^−/−^, n = 10 ), 14 days (AT1a^+/+^, n = 6; AT1a^−/−^, n = 5), 28 days (AT1a^+/+^, n = 17; AT1a^−/−^, n = 16), and 70 days (AT1a^+/+^, n = 13; AT1a^−/−^, n = 14) postischemia. The other AT1a^+/+^ mice (AT1a^+/+^ + Hyd, n = 7) were administered hydralazine (1 mg/kg/day) from 7 days before the IR operation until 70 days postischemia and were sacrificed under anesthesia to examine the influence of a decrease in SBP level on kidney injury, because the SBP levels of the AT1a^−/−^ mice were significantly lower compared to the AT1a^+/+^ mice. Sham mice were included in the experiment and sacrificed on day 3 (AT1a^+/+^, n = 3; AT1a^−/−^, n = 3), day 14 (AT1a^+/+^, n = 3; AT1a^−/−^, n = 3), and day 28 (AT1a^+/+^, n = 6; AT1a^−/−^, n = 7) postischemia in order to avoid the effects of IR and to clarify the underlying mechanism of AT1a receptor loss for the prevention of AKI-to-CKD transition. Sham operations were performed in a similar manner without the clamping of the renal pedicle.

IR, contralateral, and sham kidneys were removed, decapsulated, and immediately weighed. The kidney was cut in the transverse plane at the hilum of the kidney, and the cross-sectional area (S) was estimated using the following elliptic formula:

$${\text{S}}\;\left( {{\text{mm}}^{{2}} } \right) = {\text{maximal}}\;{\text{long}}\;{\text{axial}}\;{\text{radius}}\;\left( {{\text{mm}}} \right) \times {\text{maximal}}\;{\text{short}}\;{\text{axial}}\;{\text{radius}}\;\left( {{\text{mm}}} \right) \times \pi$$

A section of each kidney was fixed in 10% buffered formalin (Wako Pure Chemical Industries, Osaka, Japan) or methyl Carnoy’s solution for histology and immunohistochemistry, while the remainder was snap-frozen in liquid nitrogen for further analyses.

### Blood pressure

Blood pressure was measured in conscious, restrained mice via a tail-cuff apparatus (Softron BP-98A; Softron, Tokyo, Japan). SBP values were derived from an average of three measurements per animal at each time point: − 7, − 2, 0 (IR operation), 14, 35, and 70 days postischemia.

### Renal morphometric and histologic analysis

For microscopic analysis, the kidneys were dehydrated and embedded in paraffin. Sections (2-μm thick) were obtained for conventional histological assessment, including periodic acid– Schiff staining (PAS), and for immunohistochemistry. Acute tubulointerstitial injury was evaluated in 10 nonoverlapping fields forming the cortical and outer medullary areas in tissue sections stained with PAS under × 100 magnification^17^. Areas were measured with the WinRoof Image Analyzer, version 4.3.0 (Mitani, Tokyo, Japan). The degree of tubular injury in each region was expressed as a ratio relative to the entire area.

### Immunohistological analysis

An indirect immunoperoxidase method was used to identify the antigens, as previously described^16^. Macrophage infiltration was identified using rat monoclonal antibody F4/80 (1:200, BMA Biomedicals, Augst, Switzerland). Type I and III collagens were identified using rabbit polyclonal antibodies (Cedarlane Laboratories, Burlington, ON, Canada). To identify cell proliferation, rat monoclonal antibody against proliferating cell nuclear antigen (PCNA; DAKO, Carpinteria, CA, USA) was used. Aquaporin-1, as a proximal tubular marker, was identified with the use of rabbit polyclonal antibodies (Merck, Darmstadt, German). Five to ten nonoverlapping fields from the cortical and outer medullary areas in each object were examined under × 100 magnification for F4/80, Type I and III collagens, and Aquaporin-1, and under × 200 magnification for PCNA. Positively stained areas for these antibodies were measured with WinRoof and expressed as ratios to the total examined areas. Brightness and contrast were applied equally across the entire image with image editor GIMP 2.10.14. (https://www.gimp.org). Data were shown as the fold-increase or -decrease in positively stained areas in IR-kidneys compared with sham kidneys on day 3, 14, and 28 postischemia or with non-IR kidneys on day 70 postischemia.

### Real-time quantitative PCR

Total RNA was extracted from frozen kidneys and synthesis of cDNA was carried out as previously described^16^. The mRNA levels of *α-1 collagen type I, α-1 collagen type III, TGF-*β, *TNF-α*, *MCP-1, IL-18, IL-1*β, *Wnt4,* β*-catenin, AT2 receptor*, and 18S ribosomal RNA (rRNA) were measured by real-time quantitative PCR using a TaqMan Step One Plus System (Thermo Fisher Scientific, Waltham, MA, USA). The expression levels of these transcripts in each sample were normalized to 18S rRNA expression levels and were shown as the fold-increase or -decrease in mRNA expression in IR kidneys compared with sham kidneys on day 14 and day 28 postischemia or with non-IR kidneys on day 70 postischemia.

### Measurement of malondialdehyde by ELISA

Proteins were extracted from frozen kidneys, and protein concentrations were measured as described previously^16^. Renal malondialdehyde (MDA) was quantified using an MDA assay kit (ab238537, Abcam, Cambridge, MA, USA). Their values were normalized to total protein concentration.

### Measurement of renal hydroxyproline

Renal hydroxyproline was quantified using a commercially available kit (QuickZyme Hydroxyproline Assay kit, QuickZyme Biosciences B.V., Leiden, Netherlands). Their values were normalized to each kidney weight.

### Western blotting

Protein samples extracted from frozen kidneys (10 μg for HIF-1α, 20 μg for CD34) were separated by sodium dodecyl sulfate–polyacrylamide gel electrophoresis (SDS-PAGE) using NuPAGE 4%–12% Bis–Tris gels and the XCell SureLock Mini-Cell system (Thermo Fisher Scientific, Rockford, IL, USA)^46^. The proteins were separated and transferred to polyvinylidene difluoride membranes, which thereafter were blocked in Blocking One solution (Nacalai Tesque, Kyoto, Japan). Primary antibodies against HIF-1α (rabbit monoclonal; #14179; 1:1000; Cell Signaling Technology, Danvers, MA, USA) and CD34 (rabbit monoclonal; ab81289; 1:10,000; Abcam) diluted in Can Get Signal Solution (TOYOBO, Osaka, Japan) were used. To detect the expression of α-tubulin on the same membranes, a rabbit monoclonal antibody to α-tubulin (ab176560; 1:4000, Abcam) was used. A horseradish peroxidase-conjugated anti-rabbit antibody (ab97051, Abcam) was used as a secondary antibody (1:2000 for HIF-1α 1:4000 for CD34 and α-tubulin). Subsequently, chemiluminescence was detected using ECL Prime Western blotting detection reagent (GE Healthcare, Little Chalfont, UK) with a CCD camera system (Ez-Capture II, ATTO, Tokyo, Japan). Image J software from the National Institute of Health (NIH, Frederick, MD, USA) were used for quantification of the expression levels of all proteins. The ratios of HIF-1α and CD34 expression levels to α-tubulin were quantitated using Image J software. Representative blots were shown in Fig. 6b,c, and full-length blots were included in a Supplementary Information file (Supplementary Fig. S5).

### Statistical analysis

Data were expressed as mean ± SE, and statistical significance was set at P < 0.05. Following the Kruskal–Wallis test, differences among each group were identified using the Steel test, and the differences between the groups were compared using unpaired Student’s *t*-test with JMP software version 14.2.0 (SAS Institute, Cary, NC, USA).

## Supplementary Information

Supplementary Information.

## Data Availability

The data used to support the findings of this study are included within the article.
