# Supplementary material for: Angiotensin II type 1a receptor loss ameliorates chronic tubulointerstitial damage after renal ischemia reperfusion
Source: Sci Rep. 2021 Jan 13;11:982. doi: 10.1038/s41598-020-80209-0 (PMC7806698; doi:10.1038/s41598-020-80209-0)
Supplement: Supplementary file 1 — Supplementary Information. [file 41598_2020_80209_MOESM1_ESM.pdf]

Angiotensin II type 1a receptor loss ameliorates chronic tubulointerstitial damage after renal ischemia reperfusion

Short Title: Angiotensin II type 1a receptor and AKI to CKD

Yoko Fujita[1], Daisuke Ichikawa[1], Takeshi Sugaya[1], Keiichi Ohata[1], Jun Tanabe[1], Kazuho Inoue[2], Seiko Hoshino[2], Tatsuru Togo[2], Minoru Watanabe[3], Kenjiro Kimura[4], Yugo Shibagaki[1] and Atsuko Kamijo-Ikemori[1, 2, 3]

1. Division of Nephrology and Hypertension, Department of Internal Medicine, St. Marianna University School of Medicine, Kanagawa, Japan.
2. Department of Anatomy, St. Marianna University School of Medicine, Kanagawa, Japan.
3. Institute for Animal Experimentation, St. Marianna University Graduate School of Medicine, Kanagawa, Japan
4. JCHO Tokyo Takanawa Hospital, Tokyo, Japan.

Correspondence should be addressed to:
Atsuko Kamijo-Ikemori, M.D., Ph.D.
Division of Nephrology and Hypertension, Department of Internal Medicine,
Department of Anatomy, and Experimental Animals Institution, St. Marianna University School of Medicine
2-16-1 Sugao, Miyamae-Ku, Kawasaki 216-8511, Japan
Tel: +81-44-977-8111 ext. 3630, Fax: +81-44-976-7083
E-mail: a2kamijo@marianna-u.ac.jp

Additional Figure

Renal immunohistological analysis of tubulointerstitial damage at 28 days postischemia

Supplementary Figure 1.

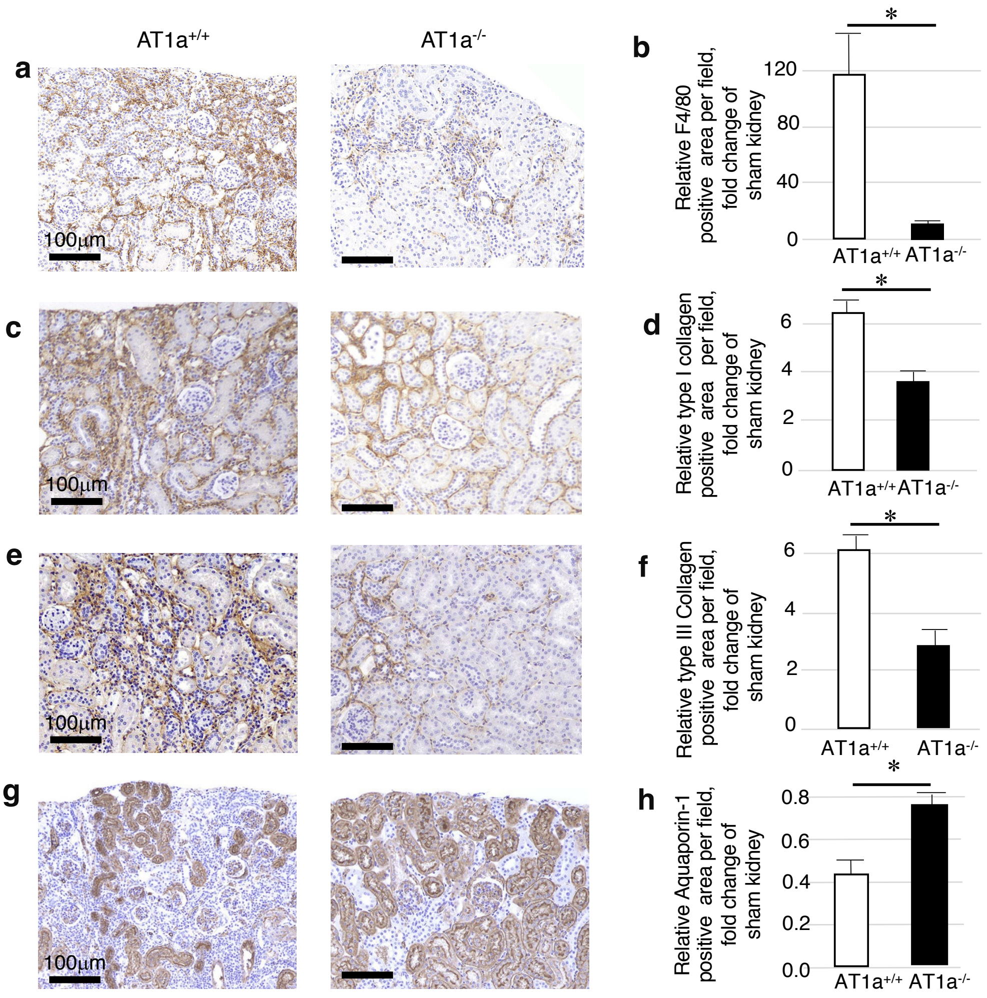

Effects of AT1a receptor loss on prevention of AKI-to-CKD transition at 28 days postischemia.

The right kidneys received unilateral ischemic reperfusion (IR) and sham mice were treated in a similar manner without clamping of the renal pedicle. (a, c, e, g) Immunohistochemical staining of F4/80 (a), type I collagen (c), type III collagen (e) and aquaporin-1 (g) in the IR-kidneys at 14 days postischemia. (b, d, f, h) Quantification of the positively stained areas of F4/80 (b), type I collagen (d), type III collagen (f) and aquaporin-1 (h) in the IR-kidneys at 14 days postischemia. The graphs show the fold-increase or -decease in each positively stained area in the IR-kidney

compared with that of the sham. Original magnification, ×100. Values are means ± SE. $^{*}P$ <0.05 vs. AT1a$^{+/+}$mice.

Gene expression analysis of p47-phox (NCF1), a subunit of NAD(P)H oxidase in the IR kidneys at 14 and 28 days postischemia

Supplementary Figure S2.

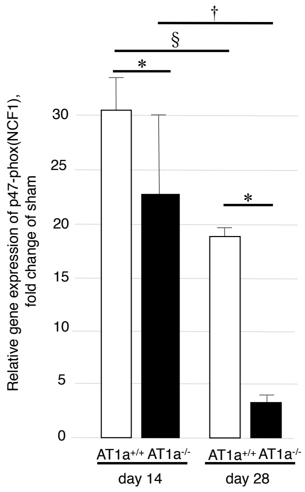

Gene expression of *p47-phox (NCF1)* in the IR kidneys at 14 and 28 days postischemia.

Total RNA was extracted from frozen kidneys and synthesis of cDNA was carried out as previously described[16]. The mRNA levels of *p47-phox (NCF1)* and 18S ribosomal RNA (rRNA) were measured by real-time quantitative PCR using a TaqMan Step One Plus System (Thermo Fisher Scientific, Waltham, MA, USA). The expression levels of the *p47-phox (NCF1)* transcript in each sample were normalized to 18S rRNA expression levels and were shown as the fold-increase or -decrease in mRNA expression in IR kidneys compared with sham kidneys at 14 and 28 days postischemia. The *p47-phox (NCF1)* gene expression levels in the IR kidneys at 14 and 28 days postischemia in wild-time mice and at 14 days postischemia in mutant mice were upregulated compared to in the kidneys of sham mice, and its levels at both 14 and 28 days postischemia were significantly lower in the AT1a$^{-/-}$ mice compared to the AT1a$^{+/+}$ mice. The *p47-phox (NCF1)* gene expression levels in the IR kidneys of both the AT1a$^{+/+}$ and the AT1a$^{-/-}$ mice was significantly decreased 28 days postischemia compared to 14 days postischemia. The graph

shows the fold-increase or -decease in gene expression in IR kidneys compared with that of the sham. Values are means ± SE. *P <0.05 vs. AT1a$^{+/+}$ mice on the same day, §P <0.05 vs. AT1a$^{+/+}$ mice at 14 days postischemia, † P <0.05 vs. AT1a-/- mice at 14 days postischemia.

Gene expression of AT2 receptor in the IR kidneys at 14 and 28 days postischemia.

Supplementary Figure S3.

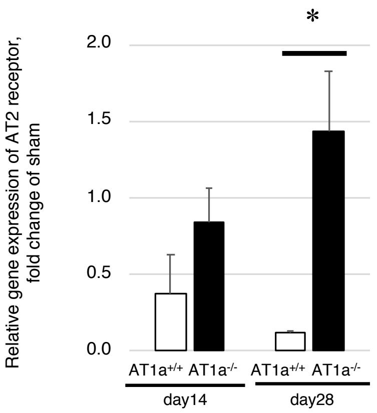

Gene expression analysis of AT2 receptor

Total RNA was extracted from frozen kidneys and synthesis of cDNA was carried out as previously described[16]. The mRNA levels of *AT2 receptor* and 18S ribosomal RNA (rRNA) were measured by real-time quantitative PCR using a TaqMan Step One Plus System (Thermo Fisher Scientific, Waltham, MA, USA). The expression levels of *AT2 receptor* in each sample were normalized to 18S rRNA expression levels and were shown as the fold-increase or -decrease in mRNA expression in IR kidneys compared with sham kidneys on day 14 and day 28 postischemia.

Although there was no significant difference in gene expression of the *AT2 receptor* in the IR kidneys at 14 days postischemia between mutant and wild-type mice, the gene expression levels of the *AT2 receptor* at 28 days postischemia were lower in the AT1a$^{+/+}$ mice compared to sham mice and were significantly lower than in the AT1a$^{-/-}$ mice. The graph shows the fold-increase or -decease in gene expression in IR kidneys compared with that of the sham. Values are means ± SE. $^{*}P$ <0.05 vs. AT1a$^{+/+}$ mice on the same day.

Acute tubular damage at 3 days postischemia in AT1a $^{+/+}$ mice, hydralazine-treated AT1a $^{+/+}$ mice, and AT1a $^{-/-}$ mice.

Supplementary Figure S4.

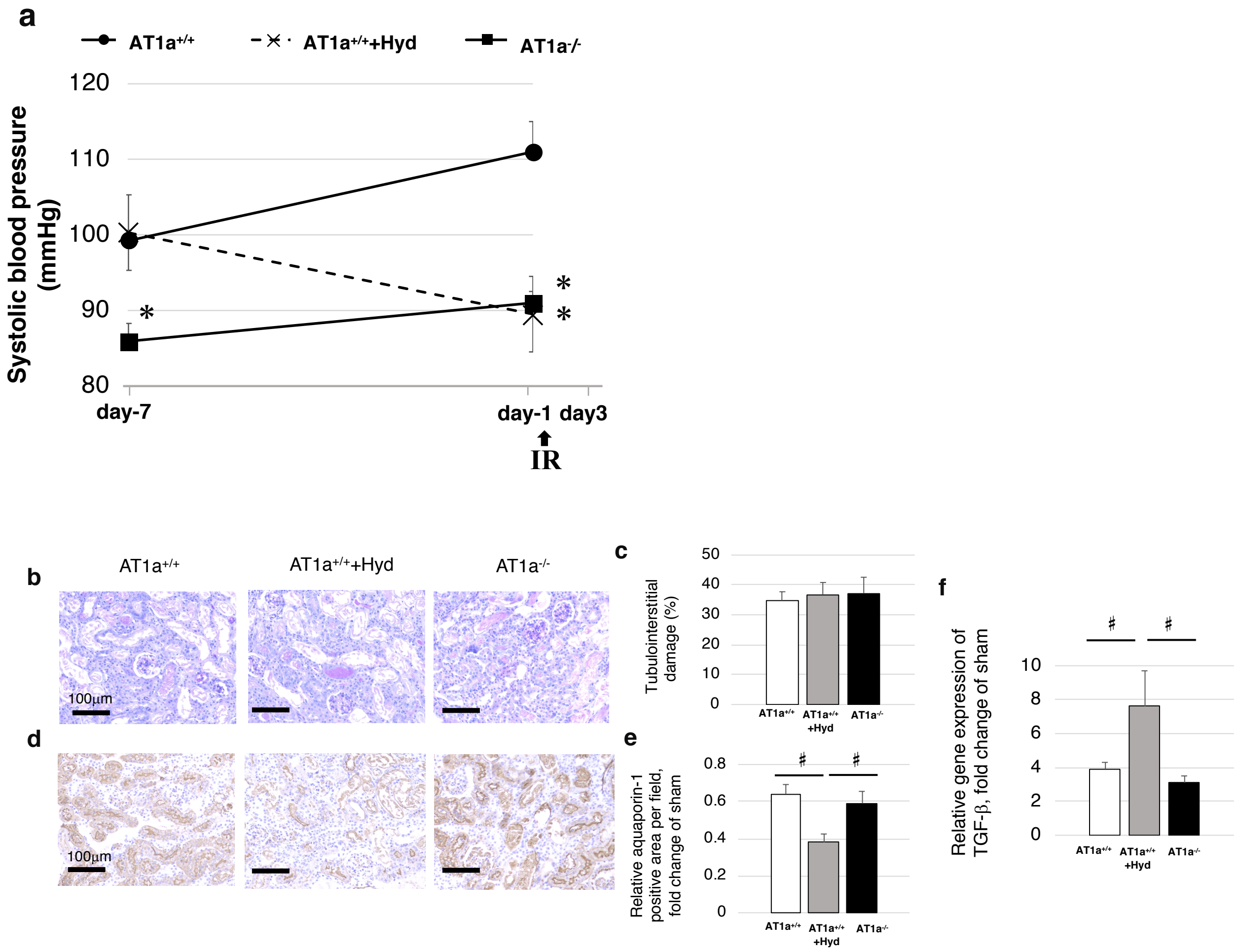

Inactivation of the AT1 receptor did not worsens acute tubular injury at 3 days postischemia in spite of their low blood pressure.

In order to evaluate the influence of hypotension on acute tubular damage after IR, we performed an experiment including hydralazine-treated AT1a$^{+/+}$ mice (AT1a$^{+/+}$, n = 10; AT1a$^{+/+}$+Hyd, n = 4, AT1a$^{-/-}$, n = 9). The AT1a$^{+/+}$+Hyd mice were administered hydralazine (1 mg/kg/day) from 7 days before the IR operation until 3 days postischemia. (a) The systolic blood pressure levels were similar for the AT1a$^{+/+}$+Hyd mice and the AT1a$^{-/-}$ mice at one day before the IR operation. (b, c, d, e, f) Acute tubular damage in tissue sections stained with PAS (b, c), renal expression of

aquaporin-1 (AQP1) in immunohistological analysis (d, e), and renal gene expression of TGF-β (f) using real-time quantitative PCR were evaluated at 3 days postischemia. While the degree of tubular injury in tissue sections stained with PAS was similar in three groups (c), the positive area of AQP-1 in the AT1a^{+}/$^{+}$+Hyd mice was significantly lower (e), and the gene expression of TGF-β in the AT1a^{+}/$^{+}$+Hyd mice was significantly higher compared to the other two groups (f). The graphs show the fold-increase or -decease in each positively stained area in the IR-kidney compared with that of the sham (e, f). Original magnification, ×100. Values are means ± SE. $^{*}P$ <0.05 vs. AT1a$^{+/+}$mice on the same day, $^{\#}P$ <0.05 vs. AT1a$^{+/+}$+Hyd mice.

Representative full-length blots of western blotting

Supplementary Figure S5.

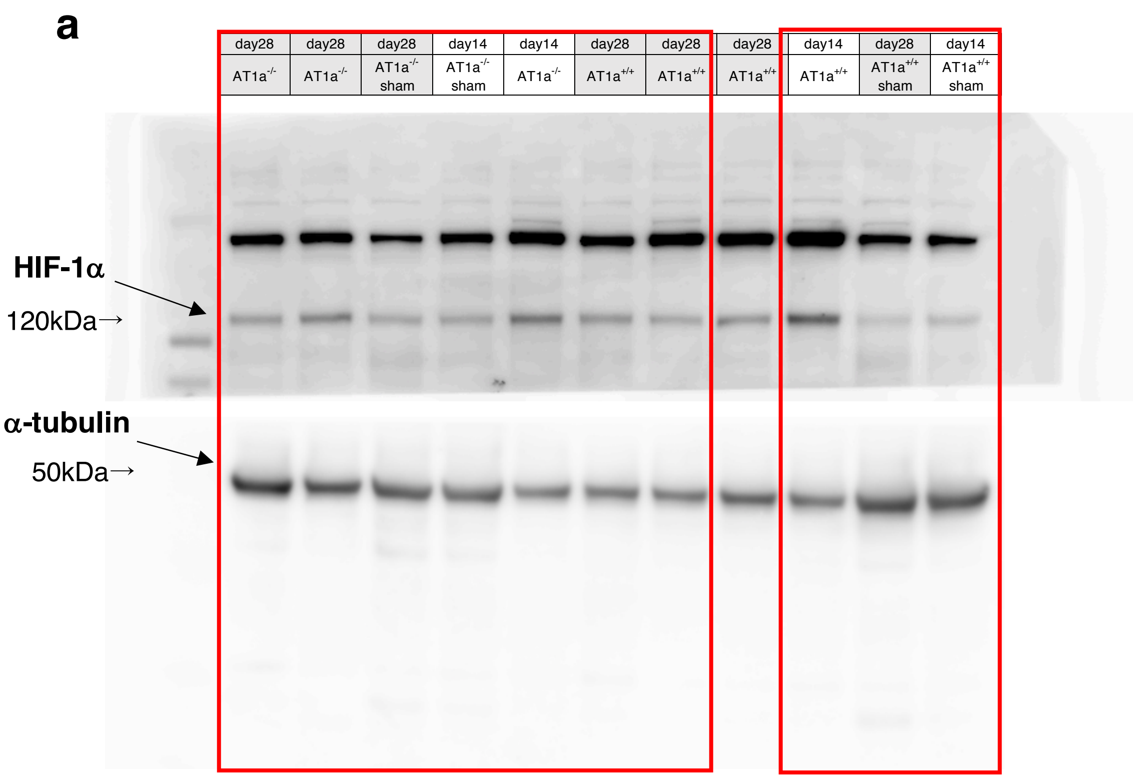

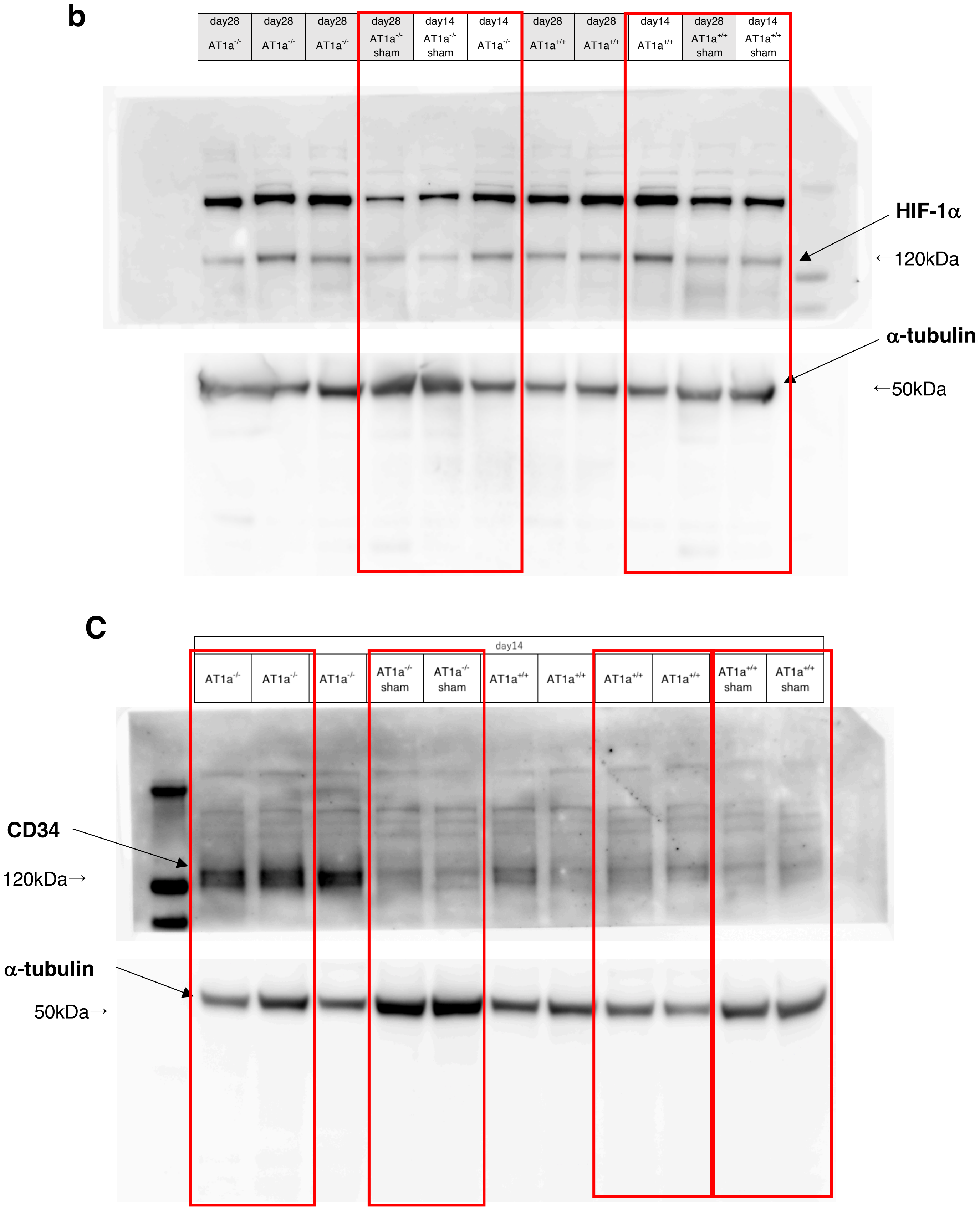

(a, b) Western blot analysis of renal HIF-1α and α-tubulin in the IR kidneys at 14 and 28

days postischemia. (c) Western blot analysis of CD34 and α-tubulin in the IR kidneys at 14 days postischemia.
